# Some Variation Factors of Freezing Point in Camel Milk

**DOI:** 10.3390/ani13101657

**Published:** 2023-05-17

**Authors:** Gaukhar Konuspayeva, Mubarak M. Al-Gedan, Fuad Alzuraiq, Bernard Faye

**Affiliations:** 1 Faculty of Biology and Biotechnology, Al-Farabi Kazakh National University, 71 Avenue Al-Farabi, Almaty 050040, Kazakhstan; konuspayevags@hotmail.fr; 2 Conservation and Genetic Improvement Center, Camel Project UTF/SAU/044/SAU, P.O. Box 761, Al-Kharj 11942, Saudi Arabia; mmnq1@hotmail.com (M.M.A.-G.); alzuraiq@gmail.com (F.A.); 3 UMR-SELMET, CIRAD-ES, Campus International de Baillarguet, CEDEX 5, 34938 Montpellier, France

**Keywords:** camel milk, freezing point, Cryostar, Milkoscan, physico-chemical properties

## Abstract

**Simple Summary:**

Freezing point is an indicator used regularly in the dairy industry to detect adulteration in cow milk, but for camel milk there is a lack of reference values. The few published references in the literature are based on few milk samples and overall, on routine analytical methods using automatic milk analyzers, which are generally not calibrated for camel milk. The present study was based on the monitoring over several months of many milk samples, analyzed with two methods, including the reference method. In addition, several potential variation factors, such as season, breed, milk composition and microbiological status, were considered. The preliminary results showed a higher freezing point of camel milk compared to cow milk published in the literature and a relatively weak correlation between the Reference and the Express method.

**Abstract:**

The freezing point degree of milk (FPD) is a classical indicator of cow milk quality. In camel milk, few references are available in the literature regarding the main factors of variation. In the present paper, two methods of FPD determination were used: the Reference method (RM) (using Cryostar) and the Express method (EM), using a milk analyzer (Milkoscan-FT1). The RM was used to determine FPD in 680 bulk raw or pasteurized camel milk samples. Regarding EM, 736 individual milk samples, 1323 bulk samples, 635 samples of pasteurized milk and 812 samples of raw milk used for cheese making were available. The variability of FPD was investigated according to month, lactation stage, milk composition, milk production and microbiological status. Correlations between methods were explored. FPD was highly correlated with most of the milk components and tended to decrease in cases of high contamination by coliforms or high total flora count. However, the weak significant correlations between the two methods indicated the necessity to specifically calibrate an automatic milk analyzer for camel milk.

## 1. Introduction

The freezing point degree (FPD) of cow milk is used in the dairy industry as an indicator of milk quality, notably to detect milk adulteration with water and to assess the amount of added water. However, other variation factors can occur, such as chemical composition, hygienic status, thermal treatment, contamination by any substance influencing the freezing, such as detergent, pollutants or antibiotics [1], or even animal factors, such as breed, physiological status, and parity [2], and farm factors, such as herd size [3]. All these variation factors have been investigated in cow milk through many analyses, leading to the creation of reference values of FPD for pure cow milk of −0.555, while the changes due to its adulteration can be easily interpreted.

In contrast, the determination of freezing point in the camel dairy industry is relatively recent, accompanying the recent development of the camel milk sector in many camel countries [4]. Some references on FPD are available in the literature, but without using the reference method (RM) ISO 5764|IDF 108:2009 [5], and most of the time, limited in the number of samples involved [6,7], or without monitoring of the same animals [8]. Usually, the determination of FPD is included in a battery of physico-chemical analyses not necessarily focused on long-term variation factors [9]. Thus, there are no referenced FPD values in camel milk based on the determination of large number of pure or adulterated milk samples. In recent years, camel milk has undergone an important development, moving from the “gift economy” to the “market economy” with a progressive integration into the modern dairy industry [10], especially in the Gulf countries [11] and Saudi Arabia [12], requiring more investment in the establishment of specific references, most notably to have a clear standard of camel milk at the national or international level [13]. Moreover, with the national price of camel milk being between 2 to 20 times higher than other milk, such as cow, sheep or goat [10], the risk of adulteration by mixing it with milk issued from these other species could be important. In such conditions, it is essential to have the right reference regarding the FPD of camel milk.

In the frame of the management in one intensive dairy camel farm, a long-term monitoring of the milk quality was achieved regarding the physico-chemical composition, including both FP and microbiological status. The present paper aimed to determine the correlations between the reference method (RM) for FPD determination with the available milk analyzers, and to study the time variability of the FP parameter in the context of intensive farming and its relationship with the microbiological status of the milk [14], its composition [15] and its processing, as well as the animal or herd status.

## 2. Materials and Methods

### 2.1. Farm Management and Origin of the Samples

All the milk samples were provided by the experimental camel farm of the “Conservation and Genetic Improvement Center”, Al-Kharj district, Riyadh, Kingdom of Saudi Arabia, during a follow-up involving 46 adult dairy camels (6–23 years old; 1–9 lactations) for 20 months. The camels belonged to 4 local breeds: Majaheem (*n* = 24), Homor (*n* = 10), Waddah (*n* = 8) and Sofor (*n* = 4). Unfortunately, the lactation rank of camels originating from outside the farm was not known (30% of the herd).

The diet of the lactating camels was composed of ad libitum alfalfa hay and 3 kg/day/animal of commercial pellets (Wafi^®^, ARASCO, Riyadh, Saudi Arabia). Camels had free access to fresh water. The milking of the animals started after a minimum of 6 weeks post-partum with a progressive introduction to the milking parlor for adaptation to the milking machine. Camels were milked twice a day after 2 months post-partum at 6 am and 4 pm in a single-tunnel milking parlor equipped with medium-pipeline (1.8 m) milking stalls and an electronic pulsator (BouMatic, Itak Company, Riyadh, Saudi Arabia). The milking machine was set at 45 kPa, 52 pulses/min and 60:40 pulsation ratio. Milking stimulation was ensured by long manual massage of the teats (almost 2 min) in the presence of the calf behind a grid according to the protocol of Ayadi et al. (2016) [16].

Two types of sampling were performed:

(i) Bulk milk samples measured daily at each milking twice a day.
(ii) Individual samples for each lactating camel once a week at the morning milking.

The total quantity of milk produced was measured daily at each milking by weighing the can and the individual quantity once a week (at the milk sampling time) with a milk recorder (Lactocorder **^®^**, Balgach, Switzerland).

### 2.2. Milk Analyses and Database

The monitoring of FP using reference method ISO 5764|IDF 108:2009 was achieved with Cryostar© (automatic I Funke Gerber, Berlin, Germany). All milk samples (bulk daily and individual weekly) were analyzed via the Express method (EM) using the automatic milk analyzer Milkoscan© (FOSS-FT1TM, Hilleroed, Denmark) calibrated for camel milk. The analyzed parameters were fat, protein, lactose, density, total solids, total non-fat, citric acid, acidity, free fatty acids and freezing point. In addition, pH and titratable acidity were determined by reference methods [17,18].

The monitoring of FP was achieved with the milk being processed into pasteurized milk, leben and cheese; the analyses also involved the milk after pasteurization, the leben and the whey. At the same time, the samples were analyzed for microbiological value according to the protocol described in Al-Rasheedi et al. (2015) [14]: milk samples (0.01 mL) were streaked on Plate Count Agar (IDF 100B, 1991) for total flora and VRBL Agar; (IDF 73B, 1998) (Merck KGaA, Darmstadt, Germany) for coliforms. The plates were then examined for colony counting.

All the data were recorded regularly on a monthly sheet, then input into the farm database written under Access© described in Al-Samghan et al. (2015) [19].

### 2.3. Statistical Analyses

As a whole, 680 milk samples were analyzed using RM. Only mixed samples were analyzed with RM (bulk milk and pasteurized milk). With EM, 736 individual milk samples and 1323 bulk samples were available. In addition, 635 samples of pasteurized milk and 812 samples of raw milk were used for cheese making. All the analyses being achieved in triplicate, the value used for statistic procedures was the mean of the 3 values.

Descriptive statistics (mean and standard deviation) were used and presented graphically. One-way ANOVA was applied for testing the time and breed effect, types of milk samples (raw, pasteurized, whey, leben), milk components or microbiological status and separately for individual and bulk data. Regarding individual data, parity was available when the camels were born in the farm (30% of the animals). Variance analysis with co-variable “parity” and “month” (ANCOVA procedure) was used on this partial table to test the breed and month effect. For assessing the microbiological effect, the values in coliforms and total flora were classified in groups as “very low values” (<10^2^), “low values” (10^2^ to <10^3^), “medium values” (10^3^ to <10^4^), “high values” (10^4^ to <10^5^) and “very high values” (<10^5^). The relationships between RM and EM data were assessed by using Pearson’s correlation. Before submitting data to ANOVA, the normality was tested by using the Shapiro–Wilk and Anderson–Darling tests. All distributions being normal, no transformation of the data occurred when applying ANOVA.

The software XLstat (Addinsoft ©, 2022, 5.1) was used for all statistical analyses.

## 3. Results

### 3.1. Correlation/Standardization of FPD Determination by RM and EM

Based on 680 milk samples for which RM was determined, the mean value was −0.332 ± 0.030 °C (range −0.260–0.478). Calculated from the 736 milk samples (individual, mixed and pasteurized milk), the mean value of EM was −0.523 ± 0.064 °C (range −0.331–0.695). However, in the farm database, 219 samples of raw mixed fresh camel milk only were simultaneously analyzed by the 2 methods. The mean value of RM in this sub-sampling was −0.321 ± 0.04, while the mean value for the same samples analyzed using EM was −0.544 ± 0.034. Thus, on average, FPD values measured with RM were higher than with EM.

**Figure 1:**
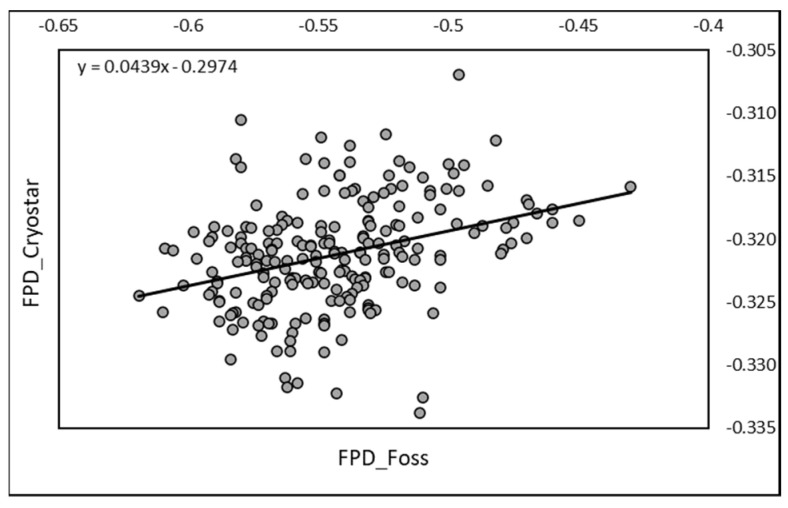
Relationship between FPD measured by RM (Cryostar) and by EM (FOSS-FT1) on raw camel milk (*n* = 219).

The correlation between RM and EM achieved on the 219 raw mixed fresh camel milk samples was positive and significant (r = 0.341; *p* < 0.0001). The equation of regression was Y = 0.0439X − 0.2974, where X was EM data (Figure 1).

**Figure 2:**
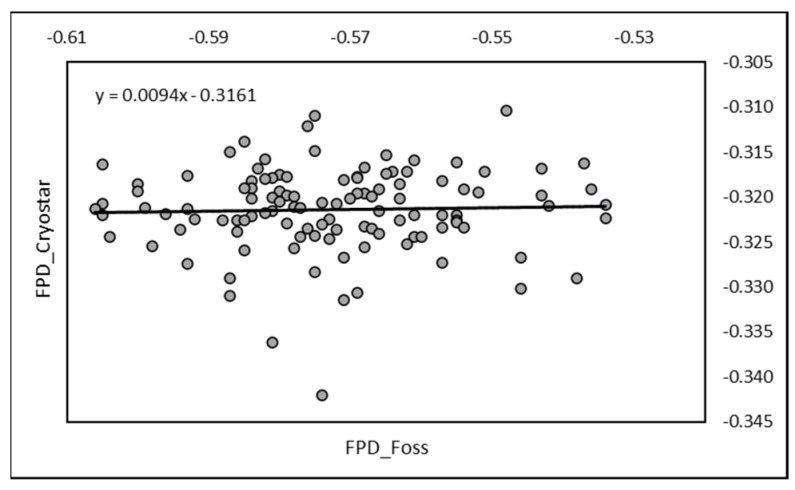
Relationship between FPD measured by RM (Cryostar) and by EM (FOSS-FT1) on pasteurized camel milk (*n* = 115).

Regarding pasteurized milk, 115 samples were simultaneously analyzed. The mean RM value was −0.321 ± 0.006 while EM value was −0.571 ± 0.019. The correlation was significant (r = 0.318; *p* < 0.001). The equation of regression was Y = 0.0094X − 0.3161 with the same parameters as the above (Figure 2).

**Figure 3:**
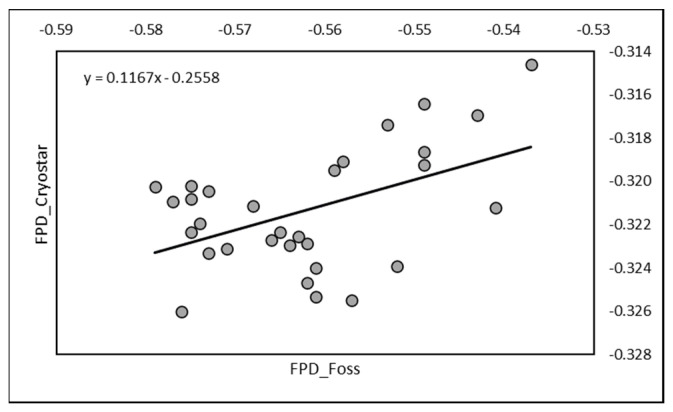
Relationship between FPD measured by RM (Cryostar) and by EM (Foss-FT1) on mixed raw milk used for cheese making.

A significant correlation (r = 0.485; *p* < 0.01) was also observed between RM (mean −0.321 ± 0.003) and EM (mean −0.562 ± 0.012) on the 30 mixed-milk samples used for cheese making. The equation of regression was Y = 0.1167X − 0.2558 (Figure 3).

By taking in account all the valuable samples (raw, pasteurized, for cheese making) analyzed simultaneously using the two methods (r = 0.172; *p* < 0.001), the equation of regression was Y = 0.6913X − 0.3476.

### 3.2. Individual Variability

**Figure 4:**
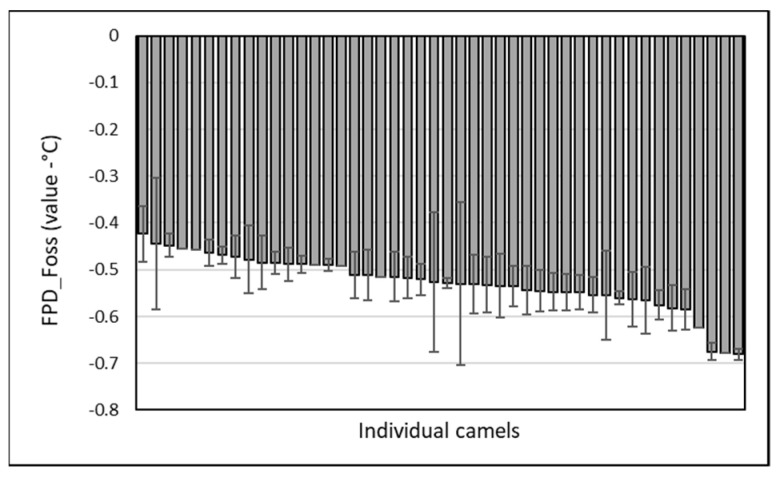
Individual variability (mean and SD) of camel milk freezing point measured with EM. The camels were sorted from higher to lower FPD values.

Throughout the period, 46 lactating camels were involved in the monitoring. FPD values from EM varied from −0.423 ± 0.060 to −0.681 ± 0.012 (Figure 4). A slight but significant breed effect (*p* < 0.05) was observed, with a higher mean value recorded for the Sofor breed (−0.464 ± 0.018) compared to the lower reported for Majaheem (−0.540 ± 0.055), with the other breeds being intermediate: Waddah (−0.515 ± 0.031) and Homor (−0.534 ± 0.069).

Due to the limited number of available data regarding parity, graphical results are not shown here, but a significant effect of parity was observed (*p* < 0.001), with optimum values at parity 5 and 6 (respectively, −0.541 ± 0.068 and −0.531 ± 0.063), while the lowest values were reported at parity 9 (−0.423 ± 0.060). There was no interaction between breed (*n* = 5) and parity (*n* = 6) levels and between breed and month (*n* = 12 levels).

### 3.3. Correlations with Milk Composition

Relationships between FPD values and milk composition were explored for individual data based on EM, and for mixed data based on both RM and EM methods.

**Figure 5:**
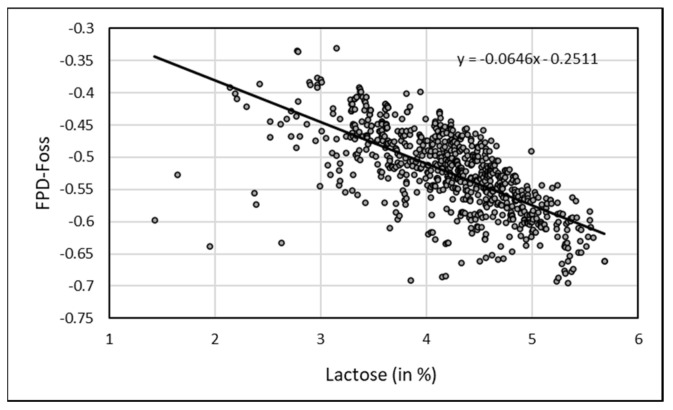
Correlation between lactose content in camel milk and FDP by EM (FDP-Foss).

Regarding individual raw camel milk, EM was positively and significantly correlated (*p* < 0.001) with all the physico-chemical parameters measured by FOSS-FT1 milk analyzer (Table 1) except for free fatty acids (FFA). The highest correlations were observed with lactose content (r = 0.684, *p* < 0.0001) and non-fat solids (r = 0.864, *p* < 0.0001) (Figure 5).

Regarding bulk camel milk, FPD by EM was also positively and significantly correlated (*p* < 0.001) to all the components of the milk (Table 2). The highest correlation was observed with lactose as for individual samples (r = 0.683; *p* < 0.0001).

Significant correlations between RM and the bulk milk composition were observed for fat, lactose, citric acid and FFA, but no significant relationship occurred with protein and density, while a negative correlation was observed with acidity (Table 3).

### 3.4. Seasonal Variation

**Figure 6:**
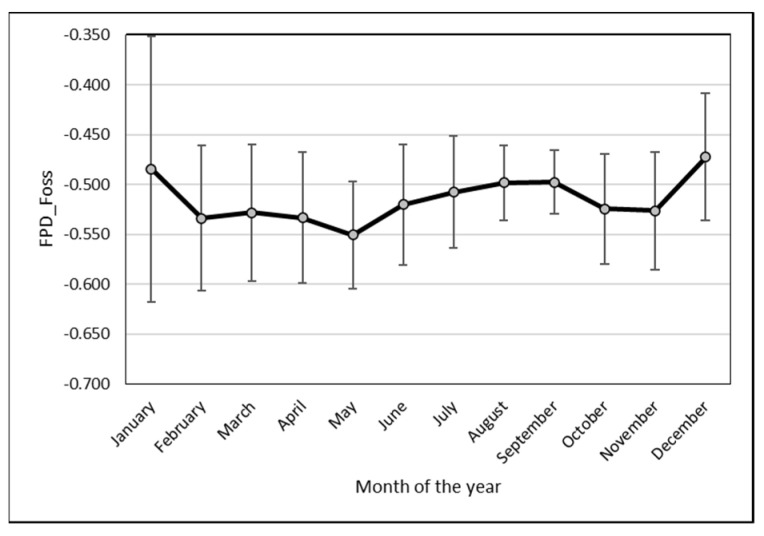
Monthly variability (mean and SD) of individual camel milk FP measured with EM.

A significant monthly variation occurred in individual EM data. The lowest value was observed in May (−0.551 ± 0.054), while the highest was reported in December (−0.472 ± 0.063). However, the differences between values in May on one hand and December and January only (Figure 6) were significant (*p* < 0.05).

**Figure 7:**
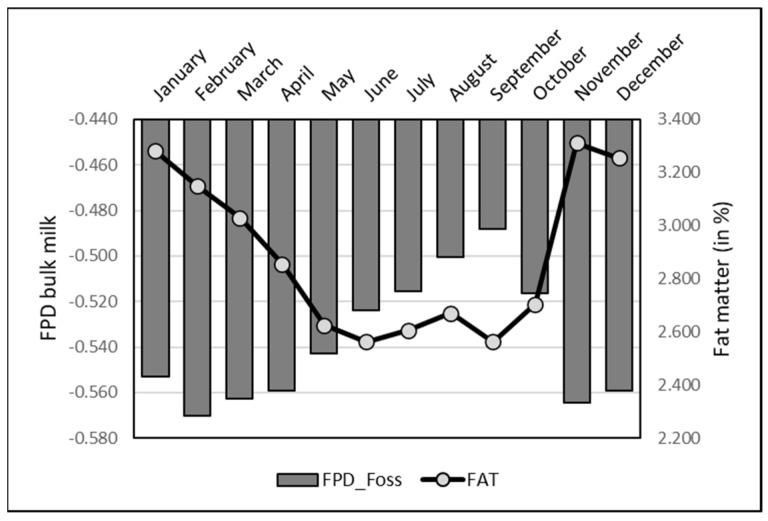
Monthly variation of FPD and fat matter (%) measured in bulk camel milk collected at each milking and based on EM data.

The monthly variation of FPD by EM on bulk raw milk was higher than for the individual records, with a pattern comparable to most of the milk physico-chemical components having seasonal variation, notably fat, protein and acidity (Figure 7). The lowest values were observed in summer, the opposite of the winter data. Indeed, correlations were significant and positive with all the physico-chemical parameters (Table 2) above. The monthly variation of FPD by EM in pasteurized milk was also similar to raw milk, with the lowest values from June to October and highest ones in winter (maximum in October: −0.508 ± 0.026; minimum in January: −0.589 ± 0.023).

Regarding FPD by RM, a monthly variation similar to EM values was observed, with a minimum in February (−0.349 ± 0.042) and maximum in July (−0.320 ± 0.006).

### 3.5. Effect of Bacteriological Status

Based on 139 available microbiological analyses, FPD by EM tended to decrease in cases of high contamination by coliforms (more than 10^5^) and of high total flora count (more than 10^6^). For coliforms, FDP by EM was −0.566 ± 0.017 in highly contaminated milk vs. −0.535 ± 0.050 in samples with low coliform count, but the difference was slightly significant (*p* < 0.05). Regarding FPD by RM, a similar trend was observed with slightly significantly lower values in groups HC and VHC.

In contrast, for total flora, no significant difference was observed between samples with count >10^6^ and count <10^3^ both for FDP by EM and RM, although a slight mean lower value was observed in group VHFT for FDP by RM (Table 4 and Table 5).

### 3.6. Effect of Milk Production

**Figure 8:**
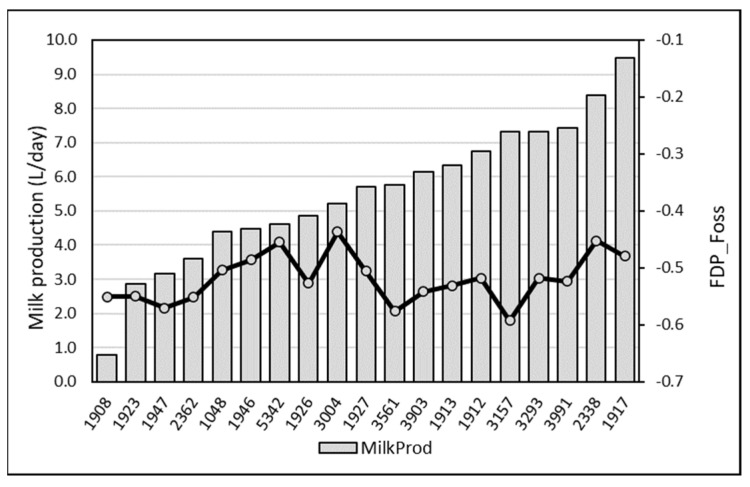
Mean daily milk production per camel and mean individual milk FDP based on the EM.

A slight but significant relationship (r = −0.140; *p* < 0.05) was observed with the milk production: FPD by EM tended to increase when the production increased. Mean FPD was −0.549 ± 0.038 when milk production was below 2 L/day vs. −0.507 ± 0.053 in case of production above 8 L/day. However, comparing the mean daily milk production per camel with the mean individual FPD by EM, this tendency was observed with the extremes only (Figure 3): the milk FDP from the lowest productive camel (n°1908) producing less than 1 L/day and from the highest productive one (n°1917) producing on average 9.5 L/day were −0.551 ± 0.039 and −0.479 ± 0.043, respectively (Figure 8).

**Figure 9:**
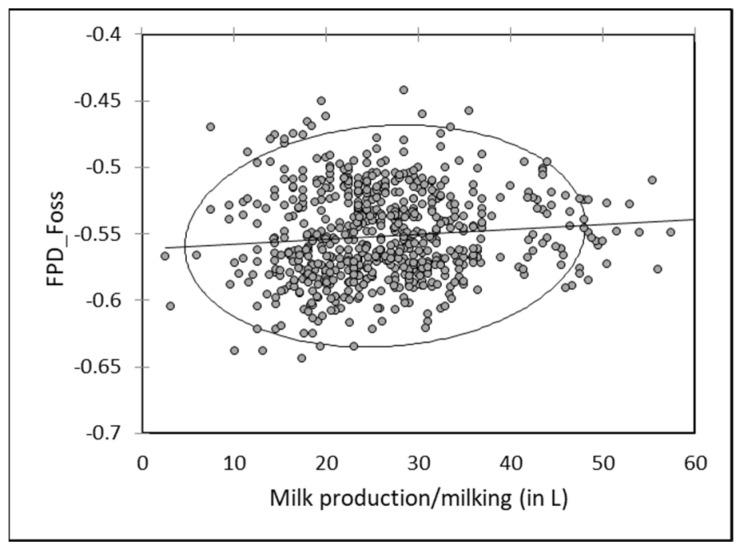
Relationship between FPD by EM (FPD_Foss) of mixed camel milk at each milking and the total amount of collected milk at the same milking (*n* = 664).

**Figure 10:**
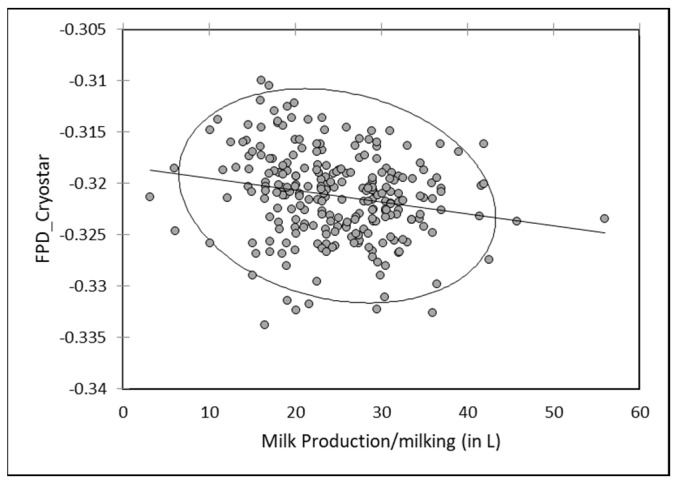
Relationship between FPD by RM (FPD_Cryostar) of mixed camel milk at each milking and the total amount of collected milk at the same milking (*n* = 250).

As for individual data, a slight negative but significant correlation (r = −0.095; *p* < 0.05) occurred between FDP by EM of milk mixed at each milking and the milk production. Freezing point increased slightly when the total production increased (Figure 9). A reverse relationship was observed between milk production and FPD by RM, with a slight tendency to observe a decrease of FPD value in bulk milk with the milk production (Figure 10).

### 3.7. Effect of Heat Treatment

The milk was pasteurized twice a week and involved the morning milking and the one from the day before. The pasteurization did not significantly modify the FDP by EM values. On average, it was −0.548 ± 0.046 for raw bulk milk vs. −0.552 ± 0.041 for pasteurized milk from the same tank. The correlation between FPD by EM of raw milk and pasteurized milk was obviously highly significant (r = 0.765; *p* < 0.0001). Similar observations were made regarding the correlations with the physico-chemical parameters of the pasteurized milk. The highest correlations were observed with the fat (r = 0.656; *p* < 0.0001), the protein (r = 0.633; *p* < 0.0001) and the lactose content (r = 0.433; *p* < 0.0001). Similarly, there was no difference between raw bulk milk and pasteurized milk FPD by RM (−0.320 ± 0.007 vs. −0.321 ± 0.007). The correlation was highly significant (r = 0.544; *p* < 0.0001).

Our data base included the composition of different types of milk (individual or mixed raw milk, pasteurized milk, mixed raw milk for cheese making, whey and fermented milk—leben). FPD was determined in most of the cases, particularly showing a slightly lower value for whey and overall for leben (Table 6).

## 4. Discussion

Using a FOSS analyzer to determine FPD in more than 4.7 million of dairy cows, Otwinowska-Mindur et al. (2017) reported a mean value of −0.5326 °C, quite comparable to the mean value obtained with the FOSS apparatus in our samples: −0.523 °C [3]. Yet, in their comparative study, Jaydeep et al. (2014) stated that with a mean value of −0.518 °C, the freezing point of camel milk was higher than cow (−0.530 °C) and buffalo milk (−0.540 °C), as well as in terms of electrical conductivity [20]. Eisa (2005) also observed lower FDP in camel milk (−0.530) compared to goat milk (−0.540) and cow milk (−0.550 °C) [21].

The Foss-FT1 apparatus used in the present study was calibrated with 161 cow milk samples, the reference method using Cryoscope [22]. The important difference between the RM and EM results could be linked to the lack of calibration with camel milk. Indeed, all the published data regarding FPD in camel milk were based on results obtained by EM [6,7,8,20] or even by using a simple thermometer [21], but never with the reference method. Moreover, none of the references regarding camel milk have specified if the used apparatus was calibrated for camel milk.

The references regarding mean FPD in camel milk varied between −0.485 to −0.621 °C (Table 7). Finally, our results regarding FPD by EM (−0.523 ± 0.064 °C) were in the range of the reported values, but according to our results acquired for the first time with the reference method, the true FPD of camel milk appeared lower than in cow milk.

Surprisingly, the range of FDP values cited by Wangoh et al. in 1998 [23] based on three camels only was regularly cited in many review papers regarding camel milk composition without critical analysis [26,27,28,29,30].

However, the quality of correlation between the two methods, probably due to the lack of FOSS calibration with camel milk, appeared low, although it was significant. Such results lead us to believe that there is an urgent need for the specific calibration of an automatic milk analyzer for camel milk. Moreover, due to the large variability in camel milk composition worldwide [31,32] and to the link between FPD and milk components, the calibration should be achieved with milk samples from different regions of the world. Indeed, in our case, despite the large number of samples, all of them came from the same farm and from a limited number of camels.

According to Henno et al. (2008) [33], in cow milk, possible effects of season and diet and their combined effect should be considered for interpreting the milk freezing point data, although Nasr et al. (2013) [6] did not observe significant difference in camel milk FPD depending on the type of pasture. However, the seasonal variability observed in our study could not be imputed to changes in the diet, as the feeding of camels was the same throughout the year, but rather to the changes in milk composition both at the individual and herd level [34]. The effect of lactation rank was not explored in our study, but no significant variation was reported in the literature [8]. At reverse, the same authors observed significant variability between camel breeds in Sudan with lower FPD values around −0.500 °C in Kenana, Daili and Arabi breeds compared to Anafi breed (−0.430 °C). A breed effect was already reported for cow milk FPD [2]. Regarding the individual variability, FPD is generally regarded as slightly heritable [35].

Nevertheless, the main factor of variation was the milk composition, the FPD values being strongly correlated with the main components of the milk as was already reported by El-Obeid et al. (2015) in camel milk [8]. The seasonal variation observed in our data base could especially be linked to the variation in fat content over the year [34]. Indeed, as camels have a seasonal reproductive cycle with calving season during winter, the lactation peak occurs in summer. Due to the dilution effect, this the season when fat and protein are minimal, contributing to an increase of FPD as it was observed in the present study. In cow milk, a significant increase of FPD was seen when the raw milk was skimmed, from −0.533 to −0.525 °C [1]. In camels receiving a diet enriched in Argan oil, FDP milk was higher (−0.582) compared to the control diet (−0.652 °C) [36].

FPD is also strongly linked to lactose and salt content in cow [31] or in mare milk [37]. Lactose and dissolved salts act significantly on FPD because their relatively high molality in milk. There is also relationships with protein content. In cow milk, Sala et al. (2010) reported an increase in the freezing point of milk with low protein content [38]. The link with protein content could be due to the structure of casein micelles, where different ions (calcium and phosphates), and more globally the mineral composition of milk may have a sensitive impact on the freezing point [39]. Brouwer (1981), for example, reported that lactose content in cow milk is responsible for 53.8% of FDP depression, potassium for 12.7%, chloride for 10.5%, sodium for 7.2% and citrates for 4.3% [40].

A high level of microbiological contamination impacting FPD is probably also due to mineral change. Indeed, the alteration of mammary epithelium in case of mastitis could lead to mineral imbalances, with an increasing level of sodium and chlorides in milk [41], which could modify the physico-chemical properties of milk, notably electric conductivity and freezing point [42]. According to Grega (1994), asymptomatic forms of mastitis decreased cow milk FPD in relation to the increasing sodium and chlorides [43].

Regarding heat treatment (pasteurization or UHT), the impact on FPD is unclear. A significant change was reported in cow milk, with a slight increase of FDP in pasteurized milk (−0.525) compared to raw milk (−0.533). At reverse, UHT treatment in cow milk did not alter FPD significantly [44].

The lower value in the FPD of whey was obviously related to the lactoserum composition of camel milk, low in protein and fat compared to milk [45]. It was reported that the addition of whey protein in adulterated milk could decrease the freezing point [46]. A depression of FPD was already described with lactose hydrolysis during the fermentation process, explaining the decrease of freezing point value in leben [47].

## 5. Conclusions

The present data, based on large number of camel milk samples, could contribute to the establishment of objective references for assessing the quality of camel milk and help to determine potential adulteration levels in raw camel milk. As FPD is sensitive to changes in ion balance, this parameter is used in cow milk to detect adulteration by adding water or milk from other species. If the higher values in camel milk determined by this reference method are confirmed in further investigations, the fraudulent addition of milk from other species, generally less expensive than camel milk, could be easily detected.

Moreover, the relative weak correlation between the EM achieved with the automatic milk analyzer (Milkoscan) without clear calibration for camel milk, and analysis with the reference method ISO 5764|IDF 108:2009 [5], requires further studies to confirm the present observed values. Although the data obtained with a non-calibrated milk analyzer allowed us to monitor the interactions with the zootechnical and physico-chemical parameters of camel milk, the present observations may be extended to more varied contexts (different farms or even different countries).

## Figures and Tables

**Table 1:** Correlation matrix between physico-chemical components of individual camel milk and freezing points measured by EM (*n* = 736). Data regarding FPD are in grey.

Parameters	FPD_EM	Fat	Protein	Lactose	Density	Acidity	CitricAc	FFA
FPD_Foss	**1**	**0.386**	**0.432**	**0.684**	**0.549**	**0.171**	**0.425**	0.050
Fat		**1**	**0.300**	**−0.237**	**−0.519**	**0.233**	−0.033	−0.001
Protein			**1**	**−0.163**	0.037	**0.877**	0.086	0.025
Lactose				1	**0.897**	**−0.390**	**0.333**	0.045
Density					**1**	**−0.170**	**0.340**	0.048
Acidity						**1**	**0.190**	0.020
CitricAcid							**1**	0.044
FFA								**1**

Bold values are different from 0 to a significance level alpha = 0.05.

**Table 2:** Correlation matrix between physico-chemical components of bulk camel milk and freezing points measured by EM (*n* = 1143). Data regarding FPD are in grey.

Parameters	FPD_Foss	Fat	Protein	Lactose	Density	Acidity	CitricAc	FFA
FPD_Foss	**1**	**0.511**	**0.611**	**0.683**	**0.469**	**0.359**	**0.427**	**0.296**
Fat		**1**	**0.325**	**−0.140**	**−0.474**	**0.259**	**0.097**	**0.537**
Protein			**1**	**0.130**	**0.272**	**0.890**	**0.223**	**0.382**
Lactose				**1**	**0.861**	**−0.124**	**0.257**	**−0.070**
Density					**1**	**0.068**	**0.251**	**−0.122**
Acidity_SH						**1**	**0.200**	**0.359**
CitricAcid							**1**	**0.186**
FFA								**1**

Bold values are different from 0 to a significance level alpha = 0.05.

**Table 3:** Correlation matrix between physico-chemical components of bulk camel milk and freezing points measured by RM (*n* = 219). Data regarding FPD are in grey.

Variables	FPD_RM	Fat	Protein	Lactose	Density	Acidity	CitricAc	FFA
FPD_cry.	**1**	**0.281**	−0.102	**0.204**	0.021	**−0.217**	**0.147**	**0.158**
Fat		**1**	**0.102**	**−0.179**	**−0.614**	**0.000**	−0.020	**0.528**
Protein			**1**	**−0.258**	**0.012**	**0.807**	**0.095**	**0.232**
Lactose				**1**	**0.811**	**−0.532**	**0.244**	**−0.118**
Density					**1**	**−0.218**	**0.250**	**−0.194**
Acidity_SH						**1**	**0.021**	**0.130**
CitricAcid							**1**	**0.129**
FFA								**1**

Bold values are different from 0 to a significance level alpha = 0.05.

**Table 4:** Camel milk composition and FPD by RM and EM value according to level of coliforms (VLCC = very low coliform count; LCC = low; MCC = medium; HCC = high and VHCC = very high).

	VLCC	LCC	MCC	HCC	VHCC	*p* Value
Fat (%)	2.758 ^a^	3.016 ^bc^	2.756 ^a^	2.784 ^ab^	3.363 ^c^	<0.001
Protein (%)	2.744 ^b^	2.718 ^b^	2.622 ^a^	2.622 ^a^	2.815 ^b^	<0.001
Lactose (%)	4.257 ^a^	4.306 ^ab^	4.423 ^ab^	4.441 ^b^	4.446 ^b^	<0.001
Acidity (SH°)	6.735 ^c^	6.558 ^bc^	6.184 ^a^	6.220 ^ab^	7.054 ^c^	<0.001
CitricAcid (%)	0.153 ^b^	0.154 ^b^	0.146 ^a^	0.148 ^ab^	0.153 ^ab^	<0.001
FFA (MevK/L)	7.744 ^a^	7.985 ^ab^	7.788 ^a^	8.430 ^b^	8.305 ^ab^	<0.05
FPD EM (°C)	−0.535 ^a^	−0.539 ^a^	−0.533 ^a^	−0.534 ^a^	−0.566 ^b^	<0.05
FPD RM (°C)	−0.317 ^a^	−0.318 ^a^	−0.319 ^ab^	−0.321 ^b^	−0.320 ^ab^	<0.05

Values within lines followed with different superscripts are significantly different.

**Table 5:** Camel milk composition and FPD by RM and EM value according to level of total flora (VLFT = very low total flora count; LFT = low; MFT = medium; HFT = high and VHFT = very high).

	VLFT	LFT	MFT	HFT	VHFT	*p* Value
Fat (%)	2.644	2.850	2.867	2.738	2.554	NS
Protein (%)	2.656	2.723	2.671	2.633	2.678	NS
Lactose (%)	4.362	4.373	4.341	4.441	4.396	NS
Acidity (SH°)	6.442 ^a^	6.617 ^a^	6.384 ^b^	6.214 ^b^	6.514 ^a^	<0.05
CitricAcid (%)	0.147	0.150	0.150	0.147	0.146	NS
FFA (MevK/L)	8.149	7.832	7.846	7.879	7.661	NS
FPD EM (°C)	−0.535	−0.541	−0.534	−0.535	−0.530	NS
FPD RM (°C)	−0.321	−0.320	−0.319	−0.320	−0.322	NS

Values within lines followed with different superscripts are significantly different. NS = Non Significant.

**Table 6:** Mean and SD of all FDP values in the farm database.

Product	N RM	FPD by RM	N EM	FPD by EM
Ind. Milk	-	-	742	−0.535 ± 0.050
Mixed milk	293	−0.320 ± 0.007	1144	−0.548 ± 0.046
Past. Milk	136	−0.321 ± 0.007	633	−0.552 ± 0.041
Whey	126	−0.332 ± 0.011	-	-
Milk for Cheese	58	−0.321 ± 0.002	812	−0.553 ± 0.044
Laben	66	−0.420 ± 0.018	-	-

**Table 7:** FPD values reported in the literature and analytical methods used.

N Samples	FPD Values	Apparatus	Reference
3 camels	−0.570–0.610	Milk analyzer	[23]
3 camels	−0.530 ± 0.008	Thermometer	[21]
8 camels	−0.518 ± 0.001	Milk analyzer	[20]
61 camels	−0.49 ± 0.04	Milk analyzer	[8]
2 herds	−0.613 ± 0.193	Milk analyzer	[24]
30 camels	−0.621 ± 0.03	Non-specified	[25]
25 camels	−0.485 ± 0.009	Digital analyzer	[7]

## Data Availability

Data are available on request via email to the corresponding author.
